# Prediction of Human Pharmacokinetics of E0703, a Novel Radioprotective Agent, Using Physiologically Based Pharmacokinetic Modeling and an Interspecies Extrapolation Approach

**DOI:** 10.3390/ijms25053047

**Published:** 2024-03-06

**Authors:** Yun-Xuan Ge, Zhuo Zhang, Jia-Yi Yan, Zeng-Chun Ma, Yu-Guang Wang, Cheng-Rong Xiao, Xiao-Mei Zhuang, Yue Gao

**Affiliations:** 1 College of Chemistry and Life Science, Beijing University of Technology, Beijing 100124, China; luckwin@yeah.net; 2 Department of Pharmacology and Toxicology, Beijing Institute of Radiation Medicine, Beijing 100850, China; zhangzhuo000@yeah.net (Z.Z.); yjy19951025@163.com (J.-Y.Y.); mazchun@139.com (Z.-C.M.); wangyg@nic.bmi.ac.cn (Y.-G.W.); xiaocr@sina.com (C.-R.X.); 3 State Key Laboratory of Toxicology and Medical Countermeasures, Beijing Institute of Pharmacology and Toxicology, Beijing 100850, China

**Keywords:** E0703, radioprotective agent, human PK prediction, physiologically based pharmacokinetic model, allometric scaling

## Abstract

E0703, a new steroidal compound optimized from estradiol, significantly increased cell proliferation and the survival rate of KM mice and beagles after ionizing radiation. In this study, we characterize its preclinical pharmacokinetics (PK) and predict its human PK using a physiologically based pharmacokinetic (PBPK) model. The preclinical PK of E0703 was studied in mice and Rhesus monkeys. Asian human clearance (CL) values for E0703 were predicted from various allometric methods. The human PK profiles of E0703 (30 mg) were predicted by the PBPK model in Gastro Plus software 9.8 (SimulationsPlus, Lancaster, CA, USA). Furthermore, tissue distribution and the human PK profiles of different administration dosages and forms were predicted. The 0.002 L/h of CL and 0.005 L of V_ss_ in mice were calculated and optimized from observed PK data. The plasma exposure of E0703 was availably predicted by the CL using the simple allometry (SA) method. The plasma concentration–time profiles of other dosages (20 and 40 mg) and two oral administrations (30 mg) were well-fitted to the observed values. In addition, the PK profile of target organs for E0703 exhibited a higher peak concentration (C_max_) and AUC than plasma. The developed E0703-PBPK model, which is precisely applicable to multiple species, benefits from further clinical development to predict PK in humans.

## 1. Introduction

Ionizing radiation (IR), a form of high-energy radiation, comprises electromagnetic waves (X-rays, γ-rays) and particles (alpha, beta, neutrons), which is powerful enough to cause the displacement of electrons from atoms and breaks in chemical bonds [1]. These forms of radiation can be released in accidents at nuclear power plants and when atomic weapons are made, tested, or used. Certain medical procedures, such as chest X-rays, computed tomography (CT) scans, positron emission tomography (PET) scans, and radiation therapy also contribute significantly. Regardless of the radiation source, exposure of human beings to radiation may lead to adverse side effects. Acute radiation syndrome (ARS), such as hematopoietic syndrome, gastrointestinal syndrome, and neurovascular syndrome, is an illness caused by the IR of the whole body or a significant part of the body by a high dose of penetrating radiation in a short period of time [2]. In addition, the World Health Organization (WHO) regarded IR as a class I carcinogen in 2017. With the wide application of IR technology like radiotherapy, protection for the population from radiation damage and exposure remains a major unmet public need, which attracts researchers’ attention in this area.

A radioprotective agent is the centerpiece of IR protection, whether pre-radiation protection or post-radiation treatment [3]. As the first Food and Drug Administration (FDA)-approved clinical radioprotective agent, Amifostine has not been widely applied to clinical therapy to date for its heterogeneous efficacy [4]. The anti-radiation effects of estrogen have also been proven [5]. Nilestriol, one kind of estriol, showed antagonistic regulation against IR-induced RNAs [6]. In addition, E0703, a new steroidal compound optimized from estradiol, significantly increased the proliferation of leucocyte, neutrophil, granulocyte, and hematopoietic progenitor cells, which ultimately improved the survival rate of KM mice and beagles in previous research [7]. However, the clinical application of E0703 was limited by the particularity of radiation protection after the completion of phase I clinical trials.

Physiologically based pharmacokinetic (PBPK) modeling and simulation is an approach that incorporates the blood flow and tissue composition of organs to define the pharmacokinetics (PK) of drugs. In recent years, the PBPK model has been widely used as a powerful tool to quantitatively delineate how certain extrinsic and intrinsic factors might influence nonproportional systemic exposures based on physiological and anatomical characteristics, as well as the physical and chemical properties of a given drug [8,9]. Alterations in PK properties, such as absorption, distribution, metabolism, and excretion (ADME), can have a substantial impact on achieving the desired therapeutic concentration of a drug. Too low a concentration results in ineffective therapy, and too high may result in side effects or even toxicity. The utilization of intelligent PBPK models and simulations presents countless opportunities for improvements in drug development. However, there are few reports on the prediction of the target organ exposure to toxic components of estrogen by the PBPK model, even if they play a significant role in IR protection and other fields.

The hydroxylation catalyzed by cytochrome P450 (CYP) enzymes is the first step in natural estradiol metabolism, which determines whether the metabolism mainly occurs in the liver. CYP1A2 and CYP3A4 were the major metabolic enzymes involved in this process with catalyzed estradiol to 2-hydroxyestradiol [10]. The metabolic mode induces weaker efficacy by oral administration than injection, which is inconvenient and uncomfortable for patients. E0703 is an optimized steroidal estrogen. By reserving cyclopentyl at the 3-position of the steroidal parent nucleus, adding β Methoxy at the 11-position, and removing α Acetyl at the 17-position, E0703 maintains oral efficacy and avoids high estrogen activity, while Enterohepatic recycling further enhances its effects. In addition, β-Cyclodextrin was added for the optimization of the drug dosage form, indicating its further benefits to in vivo drug exposure.

Currently, complete preclinical and phase I clinical trials of E0703 have finished. The pharmacokinetic and pharmacodynamic data, including Kunming (KM) mice, Rhesus monkeys, and Asian humans, have been collected, and only partial PK data are reported [7]. The purpose of this study is to use a PBPK modeling approach to predict exposures in multiple species after various routes of administration and assess its likelihood as a potential therapeutic agent for human-predicted PK profiles. To achieve this, in vitro and in vivo data for E0703 were collected on KM mice and Rhesus monkeys to understand the respective metabolism mechanisms and tissue partitioning. Then, a bottom-up combined with a top-down animal PBPK model was developed. Ultimately, a human-applicable PBPK model for multiple dosages and administration forms was established.

## 2. Results

### 2.1. Pharmacokinetic Study of E0703 in KM Mice

**Figure 1:**
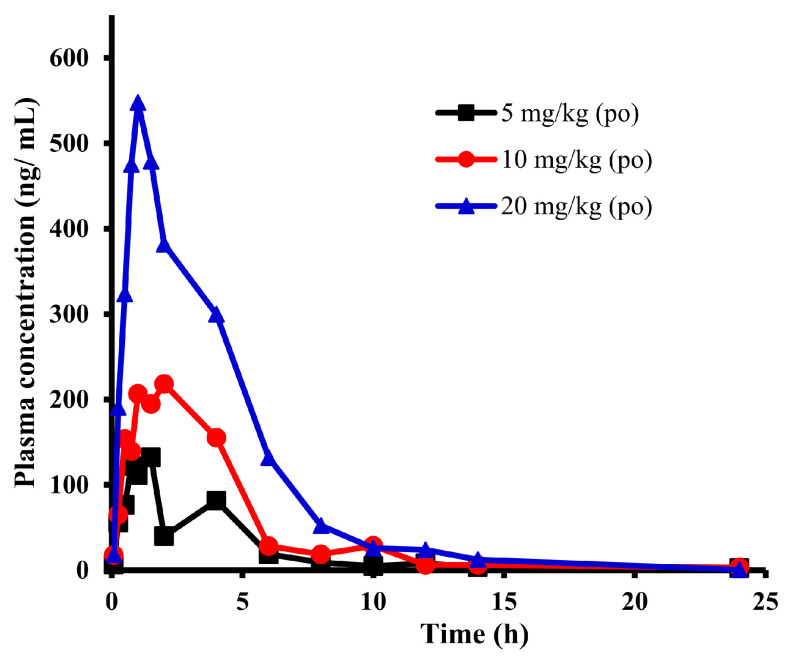
Observed time–concentration profiles of E0703 in mice after oral administration. Black, red, and blue curves represent PK profiles of 5, 10, and 20 mg/kg oral administration, respectively (*n* = 6).

The plasma concentration values at different time points of E0703 after 5, 10, and 20 mg/kg oral administration were measured, respectively. Figure 1 and Table 1 are the mean plasma drug time–concentration curves and PK parameters of E0703 in each group, and each time point is composed of six individuals (detailed plasma drug concentration and its SD values are shown in Table S1). The plasma drug concentration exhibited dose-dependent increases with similar slope of peak and elimination time curves in each group. The C_max_ of E0703 was 132.0, 218.2, and 547.7 ng/mL, with a growth ratio of 1:1.7:4.1, respectively. The AUC_(0-t)_ was 497.1, 1063.0, and 2308.8 ng × h/mL, respectively. Moreover, AUC_(0-∞)_ was 560.7, 1107.7, and 2310.8 ng × h/mL with a growth ratio of 1:2.0:4.1, respectively. Furthermore, the T_max_ was between 1 and 2 h, with no significant difference in T_1/2_, MRT, CL, and V_d_ for each group.

The above research results showed that the in vivo exposure level of E0703 increases proportionally in the dose range of 5–20 mg/kg, and C_max_ and AUC values increase linearly, while CL, T_1/2_, T_max_, and MRT have no statistically significant differences (*p* < 0.05).

### 2.2. Pharmacokinetic Study of E0703 in Rhesus Monkeys

**Figure 2:**
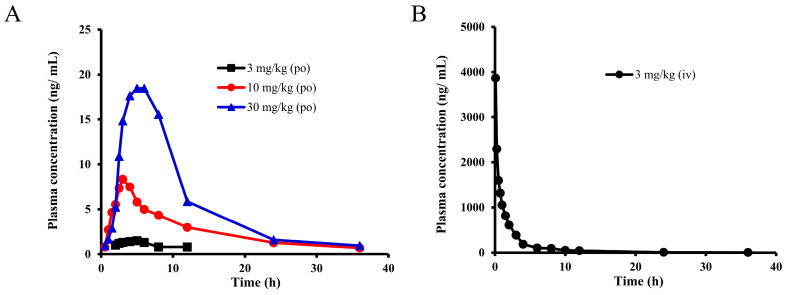
Observed time–concentration profiles of E0703 in Rhesus monkeys after oral and intravenous administration. (**A**) Black, red, and blue curves represent PK profiles of 3, 10, and 30 mg/kg oral administration, respectively (*n* = 5). (**B**) Black curve represents PK profiles of 3 mg/kg intravenous administration (*n* = 5).

The plasma concentration values at different time points of E0703 after 3, 10, and 30 mg/kg oral administration were measured, respectively. Figure 2A and Table 2 are the mean plasma drug time–concentration curves and PK parameters of E0703 in each group, and each time point is composed of five individuals (detailed plasma drug concentration and its SD values are shown in Table S2). The plasma drug concentration exhibited dose-dependent increases. The C_max_ of E0703 was 1.5, 8.4, and 18.5 ng/mL, with a growth ratio of 1:5.6:12.3, respectively. The AUC_(0-t)_ was 11.7, 93.8, and 348.8 ng × h/mL, respectively. Moreover, AUC_(0-∞)_ was 15.0, 107.9, and 370.6 ng × h/mL with a growth ratio of 1:7.2:24.7, respectively.

After 3 mg/kg intravenous administration of E0703, the mean plasma drug time–concentration curve of E0703 and PK parameters are shown in Figure 2B and Table 2 (detailed plasma drug concentration and its SD values are shown in Table S2). The C_max_ of E0703 was 3868.0 ng/mL, AUC_(0-t)_ was 4722.9 ng × h/mL, AUC_(0-∞)_ was 4802.7 ng × h/mL, CL was 2.811 L/h, and V_ss_ was 11.55 L.

For 10–30 mg/kg oral administration, C_max_ and AUC values showed a nearly linear increase with a similar CL, T_1/2_, T_max_, and MRT. In contrast, C_max_ and AUC values showed a nonlinear increase with an inconsistent CL, T_1/2_, T_max_, and MRT for 3–10 mg/kg oral administration. The slow elimination was shown in each group, with the appearance of a second absorption peak in the group of 10 mg/mL after 8–12 h oral administration, indicating the existence of hepatointestinal circulation.

### 2.3. Pharmacokinetic Study of E0703 in Asian Human

**Figure 3:**
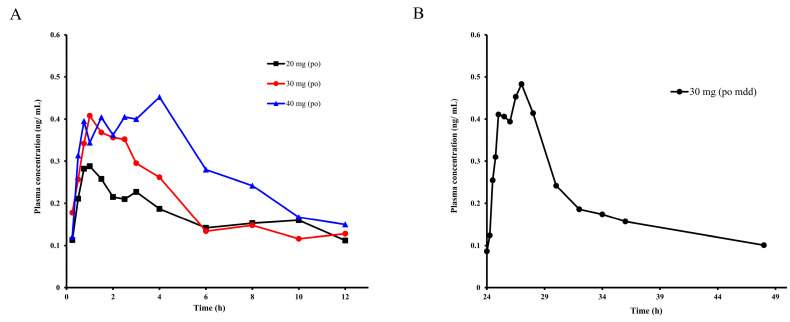
Observed concentration–time profiles of E0703 in Asian humans after single and double oral administration. (**A**) Black, red, and blue curves represent PK profiles of 20, 30, and 40 mg oral administration, respectively (*n* = 5). (**B**) Black curve represents PK profiles of 30 mg double oral administration (*n* = 5).

The plasma concentration values at different time points of E0703 after 20, 30, and 40 mg single oral administration were measured, respectively. Figure 3A and Table 3 show the mean plasma drug time–concentration curves and PK parameters of E0703 in each group, and each time point is composed of five individuals (detailed plasma drug concentration and its SD values are shown in Table S3). The plasma drug concentration exhibited dose-dependent increases. The C_max_ of E0703 was 0.29, 0.4, and 0.45 ng/mL, with a growth ratio of 1:1.4:1.6, respectively. The AUC_(0-t)_ was 2.07, 2.42, and 3.43 ng × h/mL, respectively. Moreover, AUC_(0-∞)_ was 2.70, 3.48, and 6.22 ng × h/mL with a growth ratio of 1:1.3:2.3, respectively.

The plasma concentration values at different time points of 30 mg E0703 after double oral administration were measured (Figure 3B and Table 3). Each time point is composed of five individuals (detailed plasma drug concentration and its SD values were in Table S3). The C_max_ of E0703 was 0.48 ng/mL; the AUC_(0-t)_ was 5.89 ng × h/mL; and AUC_(0-∞)_ was 8.64 ng × h/mL.

The above research results showed that the in vivo exposure level of E0703 increases proportionally in the dose range of 20–40 mg, and AUC values increase linearly.

### 2.4. Unbound Fractions of E0703 in Plasma

E0703 was tested in mouse, rat, Rhesus monkey, beagle and Asian human plasma at 37 °C for 5 h and found to be stable. A single value of unbound fractions in plasma was used for each species based on the overall mean of the data at different concentration levels (Table 4).

### 2.5. Permeability and Efflux Ratio Determination of E0703 in Caco-2 Cells

The permeability study of E0703 across the Caco-2 monolayer was performed in the direction from A to B or from B to A for 2 h. The results (Table 5) showed a low permeability of E0703 (mean P_app (A-B)_ was 1.68 × 10^−6^ cm/s) with a 2.22 efflux ratio, which indicated that E0703 was a potential efflux transport substrate. Furthermore, the low efflux ratio of E0703 may be induced by the low water solubility or cell absorption or both.

### 2.6. Liver Microsome Metabolism and CYP Enzyme Phenotype

As shown in Table 6, the fastest metabolism occurred in mouse liver microsomes, followed by monkeys, and slower metabolism occurred in humans and beagles. Comparing the liver clearance rate (CL_h_) obtained by the Well Stirred model with the liver blood flow rate (Q_h_) of various genera, it was found that if E0703 is completely metabolized and cleared by the liver, there are certain differences in the extraction of E0703 from the liver of various animal genera (CL_h_/Q_h_). The order from high to low is mice (46.6%) > humans (42.5%) > monkeys (33.9%) > beagles (29.1%).

Furthermore, recombinant human CYP enzymes displayed that CYP3A4 is the main CYP isoenzyme involved in the metabolism of E0703, with a contribution rate of 78.2%, followed by CYP1A2 and CYP2D6, with contribution rates of 12.5% and 4%, respectively (Table 7).

### 2.7. PBPK Modeling and Species Extrapolation

**Figure 4:**
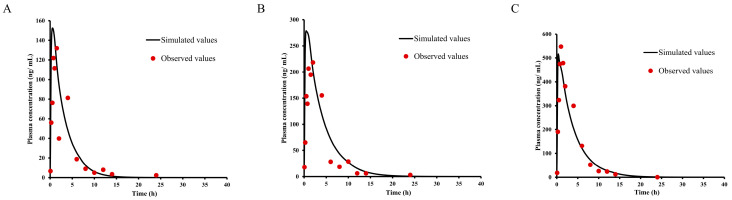
Observed and predicted time–concentration profiles of E0703 in mice. (**A**) Red points represent the concentrations obtained in mice and the black curve describes the simulated time–concentration profiles with the PBPK model of 5 mg/kg oral administration. (**B**) Red points represent the concentrations obtained in mice and the black curve describes the simulated time–concentration profiles with the PBPK model of 10 mg/kg oral administration. (**C**) Red points represent the concentrations obtained in mice and the black curve describes the simulated time–concentration profiles with the PBPK model of 20 mg/kg oral administration.

**Figure 5:**
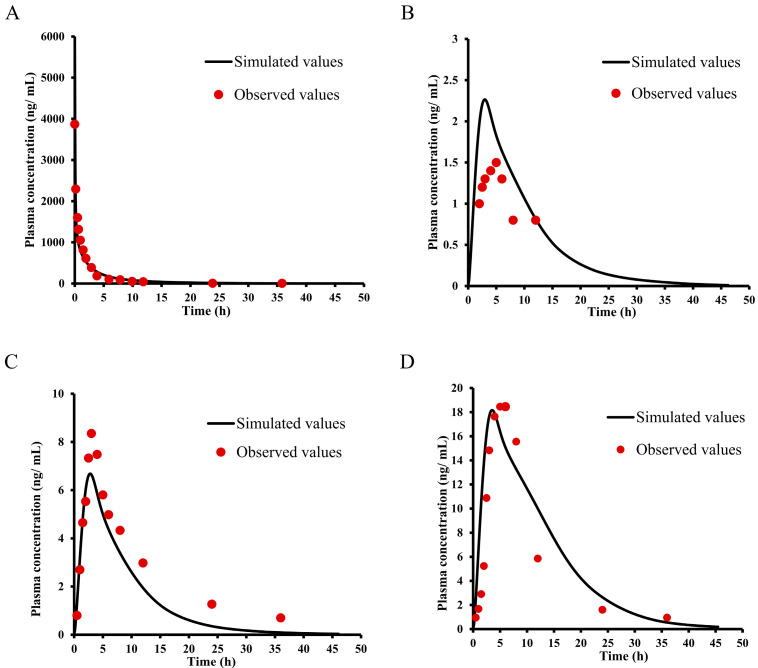
Observed and predicted time–concentration profiles of E0703 in Rhesus monkeys. (**A**) Red points represent the concentrations obtained in Rhesus monkeys and the black curve describes the simulated time–concentration profiles with the PBPK model of 3 mg/kg intravenous administration. (**B**) Red points represent the concentrations obtained in Rhesus monkeys and the black curve describes the simulated time–concentration profiles with the PBPK model of 3 mg/kg oral administration. (**C**) Red points represent the concentrations obtained in Rhesus monkeys and the black curve describes the simulated time–concentration profiles with the PBPK model of 10 mg/kg oral administration. (**D**) Red points represent the concentrations obtained in Rhesus monkeys and the black curve describes the simulated time–concentration profiles with the PBPK model of 30 mg/kg oral administration.

Based on the physicochemical properties (Table 4) and pharmacokinetic parameters of E0703, the PBPK model was initially optimized and validated in monkeys and mice by using the obtained preclinical data. For KM mice (Figure 4), the V_ss_ and CL values were estimated by related PK data. Furthermore, the existing intravenous administration PK data provided an opportunity for the verification of different tissue composition equations. The predicted V_ss_ values of monkeys by using different tissue composition equations are shown in Table 8. Using the V_ss_ values and the observed intravenous CL as inputs, simulation of the concentration–time profiles provided good approximations of the observed data (Figure 5). The predicted C_max_ and AUC values for E0703 in mice and monkeys were in the range of 0.5–2-fold of the observed, respectively. Based on these results, the same ratios of observed value to calculated value by coefficient Lokacova (Rodgers-Single) method was chosen for the human V_ss_ extrapolation (Table 8).

**Figure 6:**
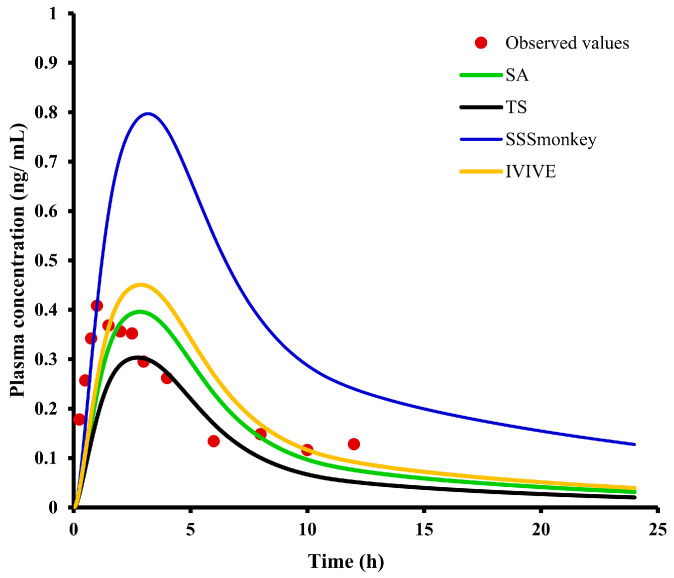
Observed and predicted time–concentration profiles of E0703 in Asian humans after 30 mg oral administration. Red points represent the concentrations obtained in Asian humans. Black, green, yellow, and blue curves describe the simulated time–concentration profiles with CL predicted by TS, SA, IVIVE, and SSS_monkey_ methods, respectively.

If CYP-mediated biotransformation is proven to be the major metabolic pathway of E0703, IVIVE with microsome/hepatocyte data is the preferred method for human clearance prediction. The human CL was predicted to be 36.09, 39.0, 21.4, and 44.5 L/h according to the in vitro-to-in vivo extrapolation (IVIVE), SA, SSS_monkey_, and TS methods, respectively. These CL estimated values were combined with the PBPK model for the comparation of human PK prediction accuracy. The predicted and observed human PK plasma concentration–time profiles and parameters after 30 mg oral administration are shown in Figure 6 and Table 9. The predicted PK parameters using CL values from IVIVE, SA, SSS_monkey_, and TS methods were within 2-fold of the observed values; the prediction values using SA methods bear the closest resemblance to the observed values.

### 2.8. Asian Human PK and Tissue Distribution Prediction

**Figure 7:**
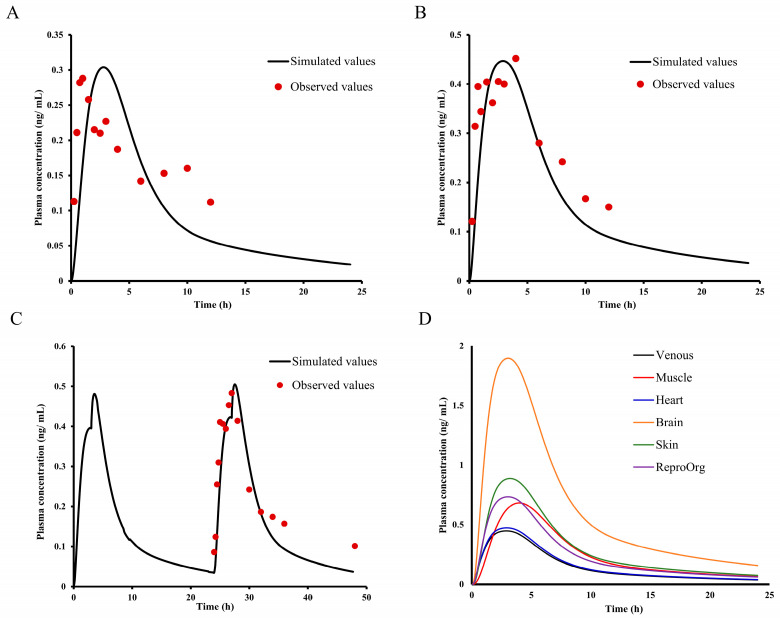
Observed and predicted time–concentration profiles of E0703 in Asian humans after 20 and 40 mg single and 30 mg double oral administration and tissue distribution prediction. (**A**) Red points represent the concentrations obtained in Asian human and the black curve describes the simulated time–concentration profiles with the PBPK model of 20 mg oral administration. (**B**) Red points represent the concentrations obtained in Asian human and the black curve describes the simulated time–concentration profiles with the PBPK model of 40 mg oral administration. (**C**) Red points represent the concentrations obtained in Asian human and the black curve describes the simulated time–concentration profiles with the PBPK model of 30 mg twice oral administration at 24 h interval. (**D**) Black, red, blue, orange, green, and purple curves describe the simulated tissue distribution in plasma, muscle, heart, brain, skin, and ReproOrg, respectively.

The CL value from the SA method was further used for the prediction of other dosages (20 and 40 mg) and double oral administration (Figure 7A–C). Overall PBPK modeling reasonably matched the plasma time–concentration profiles of E0703, with the predicted PK parameters (C_max_, T_max_, and AUC) within 2-fold of the observed values (Table 9), which verified the rationality of CL from SA methods.

Tissue distribution of several organs after 40 mg E0703 treatment was further predicted. As shown in Figure 7D, the predicted AUC values in all selected organs including brain, skin, reproductive organs (ReproOrg), muscle, and heart are higher than plasma; the brain exhibited the highest AUC. The peak time of muscle was slightly more delayed than other organs.

## 3. Discussion

E0703, a novel radioprotective agent, was synthesized for the prevention and treatment of severe acute radiation sickness. The results of a preclinical trial exhibited superior IR prevention effects with lower toxicity in KM mice and beagles [7]. Based on the clinical dosage of Nilestriol for IR protection, the proposed dose (20–40 mg, single oral administration) of E0703 was applied to the further trial. The good safety and drug tolerability shown in the phase I clinical trial indicated the considerable prospective uses for IR protection. However, further clinical trials were restricted due to the recruitment of irradiated volunteers and the pharmacodynamic trial object was replaced with Rhesus monkeys for ethics. This study aims to develop an E0703-PBPK model applied for multiple species and to assess the agent’s potential as a therapeutic agent based on the human predicted PK profiles.

To accurately describe the drug absorption process is the key step for PBPK model development. E0703 is an insoluble and lipophilic estradiol and β-cyclodextrin was added to the tablet to improve its absorption characteristics and bioavailability. The outer edge of β-cyclodextrin is hydrophilic and the inner cavity is hydrophobic, so it can provide a hydrophobic binding site, which can bind drug molecules into the heart cavity. The binding is reversible, thus increasing the water solubility of drugs insoluble in water [12]. The primary ratio between E0703 and β-cyclodextrin is 1:6, shown by the drug release curve in Rhesus monkeys (Figure S1). With the addition of the cyclodextrin module, the models had a higher fitting degree.

As the low bioavailability was observed from intravenous injection in Rhesus monkeys, a high value of the first-pass effect in the intestine was set, which only reflects the actual amount of E0703 in the liver in the body. However, for practical purposes, it may be that dissolution or absorption leads to a low amount of E0703 in the liver. After oral administration of E0703, it is absorbed into the blood by the intestinal epithelial cells. The Caco-2 cell system is a well-characterized intestinal in vitro model, frequently evaluated for the ability of chemicals to cross the intestinal barrier, as well as to study their transport mechanisms. In this study, the P_app (A-B)_ indicated the low permeability of E0703. When building the PBPK model, the value of P_app_ improves the accuracy of modeling. Furthermore, an efflux ratio over 2 implied that E0703 is a potential substrate of P-gp and E0703 has the effect of efflux transporters during intestinal absorption. Double peaks and slow metabolism existed both in the PK profiles of Asian humans and Rhesus monkeys, indicating hepatointestinal circulation. However, there is no accurate conclusion on the effect of P-gp and enterohepatic circulation for E0703 at present, so P-gp transport and hepatointestinal circulation parameters are not included in this study. The simulation results show that the predicted values of T_max_ are greater than the observed values. Moreover, the simulation PK profile of 20 mg human postprandial oral administration was the same as with fasting administration (Figure S2), with a higher C_max_ and AUC of the observed value. In combination with previous reports, it is speculated that the prediction deviation may be caused by the intestinal P-gp efflux and hepatointestinal circulation of E0703. Therefore, it is still necessary to carry out studies on the P-gp efflux and hepatointestinal circulation of E0703 to confirm this hypothesis.

Skin is the priority injured organ with a sensitive basal cell layer and capillaries for IR damage [13]. Reproductive organs, muscles, and the brain are the classical target organs for estrogen [14,15]; the effect of estrogen on the heart is also receiving increasing attention [16]. The tissue distribution prediction exhibited that all the selected organs have higher C_max_ and AUC than plasma, which indicated the wide range of anti-IR effects of E0703. The highest C_max_ and AUC in the brain also implied the risk of accumulated toxicity.

A limitation of this study was the shortage of irradiated volunteers, especially severe acute irradiated patients. Although abundant preclinical and human PK data were obtained, the accurate PK curve of E0703 after radiation is still unknown. Based on the in vitro metabolism test, CYP3A4 is the major enzyme involved in E0703 metabolism, with a contribution rate of 78.15%. Furthermore, the in vitro permeability test indicated that E0703 is the potential P-gp substrate. However, relevant reports on the effect of irradiation on drug pharmacokinetics in each species presented opposite conclusions. For instance, γ-radiation treatment increased the protein and mRNA expression in HepG2 cells [17], while the decreased protein level was evident in MDR cells [18]. The reverse influence on protein and mRNA expression of drug metabolism enzymes and transporters was also reported [18,19]. Such complicated implications affected the in vivo disposition characteristics of E0703 and increased the difficulty of PK profile description after irradiation. Furthermore, despite the fact that massive amounts of data were employed within the modeling framework, it must be admitted that there are currently limitations for the human predictive model. The precision of predicting human tissue distribution may be compromised due to the absence of validation against experimental data. In future clinical development, this PBPK model stands to be enhanced by integrating insights from an expanded range of observed preclinical and clinical pharmacokinetic studies. This iterative refinement is likely to yield a model that is not only accurate but also applicable to both healthy individuals and those exposed to radiation, thereby expanding its clinical utility.

Overall, the preclinical PK profiles of E0703 in mice and Rhesus monkeys were characterized by rapid absorption, large distribution, low clearance, and bioavailability. After the applicable predictions, CL was adopted by several in vitro prediction methods, and the human pharmacokinetics of E0703 were predicted using PBPK models with verification by the observed value. Although the current human predictive model has its limitations, the successful establishment of a human PBPK model would provide valuable insights into drug properties, allowing for the prediction of a broad range of doses. This advancement would significantly benefit the design of clinical studies, the escalation of drug doses, and the investigation of dietary impacts, as well as potential drug–drug interactions.

## 4. Materials and Methods

### 4.1. Chemicals and Reagents

E0703 (purity of 99.96%) and the internal standard IS 1229(purity > 99%) were obtained from the Beijing Institute of Radiation Medicine (Beijing, China). LC/MS-grade acetonitrile and formic acid were purchased from Fisher Scientific (Fisher Scientific, Fair Lawn, NJ, USA). By 0.1 μm filtration, deionized water was prepared with a Milli-Q purification system (Millipore, Milford, MA, USA).

### 4.2. PK Studies in KM Mice, Rhesus Monkeys, and Asian Human

All of the animals were supplied by Military Medical Research Institute. Protocols for the care and handling of animals were in accordance with the procedures approved by the Animal Care and Use Committee of Military Medical Research Institute (Beijing, China). The human PK study was approved by the Medical Ethics Committee of No. 302 Hospital of PLA (Beijing, China), and was given written informed consent for participation in the clinical trials, according to the principles of the Declaration of Helsinki and Good Clinical Practice. KM mice and Rhesus monkeys were fasted for 12 h before administration. E0703 was dissolved in medical oil for mouse oral administration and monkey intravenous administration, with a tablet used for monkey oral administration. The sampling type for all species was serial sampling.

Two hundred and seventy KM mice (18–22 g, 3–4 weeks old, three males and three females in each group) were administered an oral dose of 5, 10, or 20 mg/kg E0703. Blood samples (approximately 0.5 mL) were collected in heparin-coated tubes per dose at 0, 5, 15, 30, and 45 min, 1, 1.5, 2, 4, 6, 8, 10, 12, 14, and 24 h post-dose. Plasma was prepared by centrifugation at 5000× rpm for 10 min and the samples were then stored at −80 °C before analysis.

Male and female Rhesus monkeys (4–5 kg) were treated by 3 mg/kg intravenous administration or 3, 10, or 30 mg/kg oral administration (*n* = 5). Blood samples (approximately 1 mL) were collected in heparin-coated tubes per dose at 0, 5, 15, 30, and 45 min, 1, 1.5, 2, 3, 4, 6, 8, 10, 12, 24, and 36 h post-dose for intravenous administration and per dose at 0, 15, and 30 min, 1, 1.5, 2, 2.5, 3, 4, 5, 6, 8, 12, 24, 36, and 48 h post-dose for oral administration. Plasma was prepared by centrifugation at 1500× *g* for 10 min and the samples were then stored at −80 °C before analysis.

The human PK study was conducted with 20/30/40 mg single oral administration, 30 mg double oral administration at 24 h intervals, and 20 mg postprandial administration of E0703 to healthy human volunteers (*n* = 5), respectively. The simulated plasma concentration–time curves and the predicted PK parameters were compared with the observed human data to evaluate the accuracy of prediction.

### 4.3. E0703 Analysis in KM Mice, Rhesus Monkeys, and Asian Human Plasma Samples

E0703 analysis for KM mice, Rhesus monkeys, and Asian humans is consistent with the reports from Gao’s lab [7].

### 4.4. Non-Compartmental PK Analysis

The plasma concentration data of E0703 in each species were analyzed by non-compartmental analysis with WinNonlin 5.21 software (Pharsight Corporation, Mountain View, CA, USA).

### 4.5. Permeability of E0703 in Caco-2 Cells

Human colon carcinoma (Caco-2) cells, which were obtained from Military Medical Research Institute, were maintained in Dulbecco’s modified Eagle’s medium. Caco-2 cells were seeded onto polyethylene membranes (PET) in 96-well Falcon insert systems at 2 × 10^5^ cells/cm^2^ for 21–28 days for confluent cell monolayer formation. The medium was changed every 3–4 days. Test compounds were diluted with the transport buffer (HBSS without BSA) from a 10 mM stock solution to a concentration of 10 µM and applied to the apical or basolateral side of the cell monolayer. Permeation of the test compounds in the direction from A to B or from B to A was determined in duplicate over a 120-min incubation at 37 °C and 5% CO_2_ with a relative humidity of 95%. In addition, the efflux ratio of each compound was also determined. Test and reference compounds were quantified by LC-MS/MS analysis based on the peak area ratio of analyte/IS.

The apparent permeability coefficient P_app_ (cm/s) was calculated using the following equation:

P_app_ = (dCr/dt) × Vr/(A × C_0_)

where dCr/dt is the cumulative concentration of the compound in the receiver chamber as a function of time (S); Vr is the solution volume in the receiver chamber (0.1 mL on the apical side, 0.25 mL on the basolateral side); A is the surface area for the transport, i.e., 0.0804 cm^2^ for the area of the monolayer; C_0_ is the initial concentration in the donor chamber.

The efflux ratio was calculated using the following equation:

Efflux Ratio = P_app_ (B to A)/P_app_ (A to B)

Percent recovery was calculated using the following equation:

% Recovery = 100 × [(Vr × Cr) + (Vd × Cd)]/(Vd × C_0_)

% Total recovery =100 × [(Vr× Cr) + (Vd × Cd) + (Vc × Cc)]/(Vd × C_0_)

where Vd is the volume in the donor chambers (0.1 mL on the apical side, 0.25 mL on the basolateral side); Cd and Cr are the final concentrations of transport compound in donor and receiver chambers, respectively. Cc is the compound concentration in the cell lysate solution. Vc is the volume of insert well (0.1 mL in this assay).

### 4.6. Plasma Protein Binding

The extent of protein binding by 1 µM E0703 was determined by equilibrium dialysis in the plasma of KM mice, Sprague–Dawley rats, beagles, Rhesus monkeys, and Asian humans. E0703 was prepared in DMSO as a stock solution and then diluted with blank plasma to achieve the test concentrations. Equilibrium dialysis was performed with a 96-well device. Then the entire apparatus was placed in a shaker (60 rpm) for 5 h at 37 °C. After incubation, an aliquot of 25 µL from both the donor sides and receiver sides of the dialysis apparatus was placed in new sample preparation plates and the aliquots were mixed with same volume of opposite matrixes (blank buffer to plasma and vice versa). The samples were quenched with 200 μL MeOH containing internal standard (IS). All the samples were vortexed (from 0 h and 5 h) at 600× rpm for 10 min followed by centrifugation at 6000× rpm for 15 min. A total of 100 μL of the supernatant was transferred from each well into a 96-well sample plate containing 100 μL of MeOH for LC/MS analysis.

### 4.7. In Vitro Metabolism of E0703

The reaction system between E0703 and liver microsomes was optimized by E0703 concentration, microsomal protein content, and incubation time. LC-MS/MS was used for the quantitative determination of the residual amount of E0703. The relative production percentage of E0703 metabolites in all species of liver microsomes was analyzed and compared. Also, the corresponding metabolic kinetic parameters were calculated and compared.

The equation [20] was used as follows:

T_1/2_ = −0.693/*κ*

$$Cl_{int}=\frac{0.693}{T_{1/2}}\times \frac{incubation(mL)}{liver(mg)}\times \frac{microsomes(mg)}{liver(mg)}\times \frac{liver(mg)}{BW(kg)}$$

$$Cl_{h}=\frac{Q_{h}\times Cl_{int}}{Q_{h}\times Cl_{int}}$$

Recombinant human CYP isoenzymes (CYP1A2, CYP2A6, CYP2C9, CYP2C19, CYP2D6 and CYP3A4) were used for in vitro metabolism experiments. Residual E0703 was determined by LC-MS/MS, and the metabolic degree of CYP450 enzyme subtype on E0703 was evaluated using total normalized rate (TNR) method.

The equation [20] was used as follow:

$$\%\mathrm{TNR}=\frac{\mathrm{pmol}/\min/\mathrm{pmol}\;\mathrm{rCYP}n\times \mathrm{pmol}\;\mathrm{mCYP}n/\mathrm{mg}}{\sum \left(\mathrm{pmol}/\min/\mathrm{pmol}\;\mathrm{rCYP}n\times \mathrm{pmol}\;\mathrm{mCYP}n/\mathrm{mg}\right)}\times 100$$

### 4.8. Prediction of CL Using Simple Allometry (SA) Method with Body Weight

The equation [20] was used as follows:

CL= a × (BW)^b^

where a and b are the coefficient and scaling exponent, respectively, and BW is the body weight.

### 4.9. Prediction of CL_human_ from Single-Species Allometric Scaling (SSS_monkey_)

The equation [20] was used as follows:

CL_human_ = CL_animal_ × (BW_human_/BW_animal_)^0.75^

### 4.10. Prediction of CL_human_ Using the Two-Species Scaling Method (TS)

The equation [21] was used as follows:

CL_human =_ α_rat-monkey_ × (W_human_)^0.650^

where α_rat-monkey_ is the coefficient obtained from conventional allometric scaling of the two-species data and 0.650 is a fixed value that was obtained and optimized as the allometric exponent.

### 4.11. Prediction of Plasma Concentration–Time Profiles Using the GastroPlus PBPK Model

All modeling parameters were imported into Gastro Plus software 9.8 (SimulationsPlus, Lancaster, CA, USA). An advanced atrioventricular absorption and transport (ACAT) model was used to simulate the time–concentration curve [22]. The drug distribution between tissue and blood was assumed to be a perfusion-limited model. The liver and kidney were considered to be the only sites of elimination.

The estimated methods of tissue-to-plasma partition coefficients (K_p_ values) were available in Gastro Plus software 9.8 and the Lokacova (Rodgers-Single) method was selected for further modeling. Another key modeling parameter is CL and the observed in vivo CL was obtained from intravenous administration of Rhesus monkeys. For PBPK modeling in KM mice, CL was estimated from PK data. For PBPK modeling in Asian humans, CL values were predicted from four in vivo allometric methods (TS, SA, IVIVE, and SSS_monkey_ methods).

### 4.12. Statistical Analysis and Model Evaluation

For accuracy assessment of a model simulation, acceptance is set to be within 2-fold. For comparison between groups, the statistically significant difference is set for a *p*-value of less than 0.05 using analysis of variance, with the Student’s *t*-test.

## 5. Conclusions

In this study, amounts of measured physiology and PK parameters about E0703 were collected in the first stage, and suitable fit values were added for lacking parameters. After intravenous and oral PBPK models were established and optimized in mice and Rhesus monkeys, the accuracy of the E0703-PBPK model was verified with multiple doses. Further, several in vivo methods were used to predict human CL and the observed value was used to evaluate the accuracy of each method. Finally, the PBPK models were used for predicted tissue distribution, different dosages, and administration forms of E0703. To summarize, although the current human predictive model has its limitations, the development of the E0703-PBPK model provides an appropriate tool for in vivo E0703 studies, assesses its likelihood as a potential therapeutic agent by the human predicted PK profiles, and supports further E0703 mechanism research.

## Figures and Tables

**Table 1:** Observed PK parameters in mice treated by oral administration of E0703 (5/10/20 mg/kg).

Parameter	5 mg/kg	10 mg/kg	20 mg/kg	Units
C_max_	132.0	218.2	547.7	ng/mL
T_max_	1.5	2	1	h
T_1/2_	18.37	10.00	2.28	h
AUC_(0-t)_	497.1	1063.0	2308.8	ng × h/mL
AUC_(0-∞)_	560.7	1107.7	2310.8	ng × h/mL
(CL/F)/kg	8.92	9.03	8.66	L/h/kg
MRT	9.60	5.44	3.78	h
(V_ss_/F)/kg	85.61	49.12	32.73	L/kg

**Table 2:** Observed PK parameters in Rhesus monkeys treated by intravenous administration (3 mg/kg) and oral administration of E0703 (3/10/30 mg/kg).

Label	3 mg/kg	10 mg/kg	30 mg/kg	3 mg/kg, iv	Units
C_max_	1.5	8.4	18.5	—	ng/mL
T_max_	5	3	6	—	h
T_1/2_	2.86	13.96	15.96	13.17	h
AUC_(0-t)_	11.7	93.8	348.8	4722.9	ng × h/mL
AUC_(0-∞)_	15.0	107.9	370.6	4802.7	ng × h/mL
Bioavailability	0.35	0.76	0.87	—	%
CL	—	—	—	2.811	L/h
(CL/F)/kg	200.4	92.69	80.95	—	L/h/kg
MRT	8.23	17.06	17.50	4.11	h
V_ss_	—	—	—	11.55	L
(V_ss_/F)/kg	1649.8	1581.6	1417.0	—	L/kg

**Table 3:** Observed PK parameters in Asian humans treated by oral administration of E0703 (20/30/40 mg) and double oral administration (30 mg).

Label	20 mg	30 mg	40 mg	30 mg, mdd	Units
C_max_	0.29	0.40	0.45	0.48	ng/mL
T_max_	1	1	4	27	h
T_1/2_	3.89	5.69	12.91	18.86	h
AUC_(0-t)_	2.07	2.42	3.66	5.89	ng × h/mL
AUC_(0-∞)_	2.70	3.48	4.11	8.64	ng × h/mL
(CL/F)/kg	105.8	123.3	91.83	99.26	L/h/kg
MRT	8.24	9.39	16.55	45.18	h
(V_ss_/F)/kg	871.0	1157.9	1520.1	4484.1	L/kg

**Table 4:** Physicochemical parameters of E0703 used for PBPK models.

Parameter	Value	Source
Molecular weight (g/mol)	370.54	Measured in this study
Log P	5.13	Gastro Plus 9.8
Solubility at pH 7 (mg/mL)	5 × 10^−5^	Assessment value
P_app_ (cm/s)	1.68 × 10^−6^	Measured in this study
F_u,p_ in mouse, rat, Beagle, Rhesus monkey, and human plasma (%)	1.3, 0.7, 13, 1.2, 0.9	Measured in this study
R_b/p_ in mice, Rhesus monkeys and Asian humans	0.82	Gastro Plus 9.8

**Table 5:** Permeability of E0703 in Caco-2 cells.

Compound	Mean P_app_ (10^−6^ cm/s)		Efflux Ratio	Mean Recovery %		Mean Total Recovery %	
	A to B	B to A		A to B	B to A	A to B	B to A
Atenolol	0.73	—	—	90.9	—	—	—
Propranolol	29.38	—	—	81.0	—	—	—
Digoxin	0.65	14.64	22.48	101.3	95.3	—	—
E0703	1.68	3.74	2.22	27.8	61.1	43.7	62.7

**Table 6:** Metabolic clearance rate and half-life of E0703 in mouse, Rhesus monkey, beagle, and Asian human liver microsomes.

Species	T_1/2_ (min)	Cl_int_ (mL/min/kg)	CL_h_ (mL/min/kg)	Q_h_ ^1^ (mL/min/kg)
Beagle	157.5	12.7	9	30.9
mouse	69.3	78.8	42	90
Rhesus monkey	83.5	22.4	14.8	43.6
Asian human	105	15.3	8.8	20.7

^1^ Hepatic blood flow [11].

**Table 7:** Effect evaluation of each CYP enzyme to E0703 metabolism by normalization method.

rCYP	Metabolic Rate pmol/min (pmolrCYP)	CYPs Content pmol/mg (Protein)	Normalized Metabolic Rate pmol/min mg (Protein)	Ratio (%)
1A2	0.58	45	25.95	12.49
2C9	0.06	96	5.33	2.57
2C19	0.09	19	1.71	0.82
2A6	0.06	68	4.08	1.96
2D6	0.83	10	8.3	4
3A4	1.5	108	162.33	78.15

**Table 8:** The predicted V_ss_ values of Rhesus monkeys by using different tissue composition equations.

Methods	Poulin and Theil	Berezhkovskiy	Rodgers, Leahy, Rowland	Lokacova (Rodgers-Single)
Values (L)	28.64	21.78	26.77	26.77

**Table 9:** Predicted and observed PK parameters in Asian humans treated by oral administration of E0703 (30 mg).

Label	Observed	IVIVE, SA, SSS_monkey_, TS	Units	Fold Error (O/S)
C_max_	0.40	0.45/0.4/0.8/0.3	ng/mL	0.89/1.00/0.50/1.33
AUC_(0-t)_	2.42	3.65/3.14/7.8/2.29	ng × h/mL	0.66/0.77/0.31/1.06
AUC_(0-∞)_	3.48	4.25/3.6/10.5/2.58	ng × h/mL	0.82/0.97/0.33/1.35

## Data Availability

The data presented in this study are available on request from the corresponding author (accurately indicate status).

## Supplementary Materials

The following supporting information can be downloaded at: https://www.mdpi.com/article/10.3390/ijms25053047/s1.
