# Supplementary material for: Prediction of Human Pharmacokinetics of E0703, a Novel Radioprotective Agent, Using Physiologically Based Pharmacokinetic Modeling and an Interspecies Extrapolation Approach
Source: Int J Mol Sci. 2024 Mar 6;25(5):3047. doi: 10.3390/ijms25053047 (PMC10931676; doi:10.3390/ijms25053047)
Supplement: Supplementary file 1 [file ijms-25-03047-s001.zip › Figure S.pdf]

Figure legends

Figure S1. Release curve of E0703 tablets in Rhesus monkeys. Black, red and blue curves represent PK profiles for the 1:1, 1:3, 1:6 of the addition ratio of β-cyclodextrin, respectively (*n* = 5).

Figure S2. Observed and predicted time–concentration profiles of E0703 in Asian humans after 20 mg postprandial oral administration. Red points represent the concentrations obtained in Asian human and the black curve describes the simulated time–concentration profiles with the PBPK model of 20 mg postprandial oral administration.
