# Supplementary figures and images for: Prediction of Human Pharmacokinetics of E0703, a Novel Radioprotective Agent, Using Physiologically Based Pharmacokinetic Modeling and an Interspecies Extrapolation Approach
Source: Int J Mol Sci. 2024 Mar 6;25(5):3047. doi: 10.3390/ijms25053047 (PMC10931676; doi:10.3390/ijms25053047)

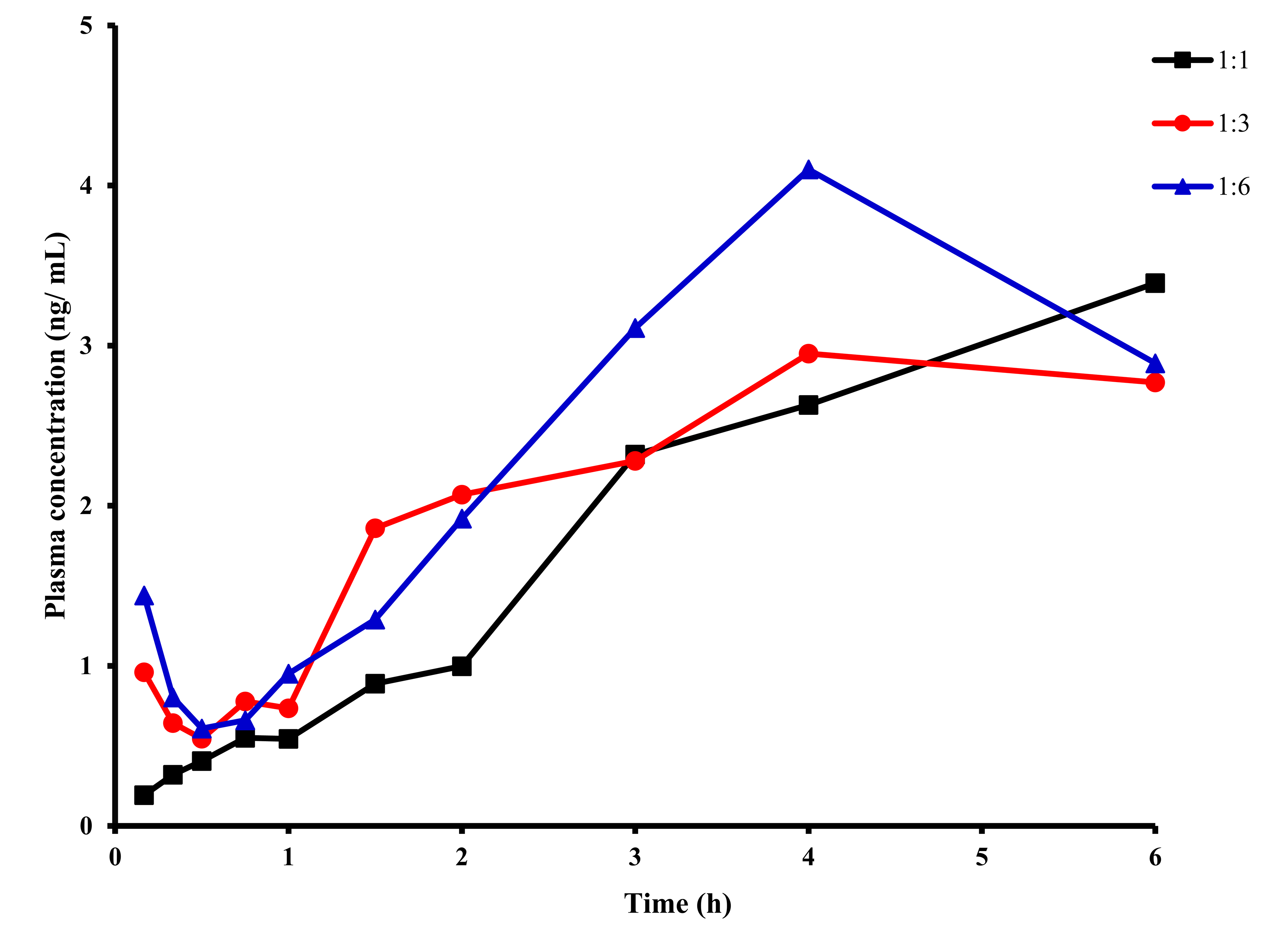

Supplement: Supplementary file 1 [file ijms-25-03047-s001.zip › Figure S1.tif]

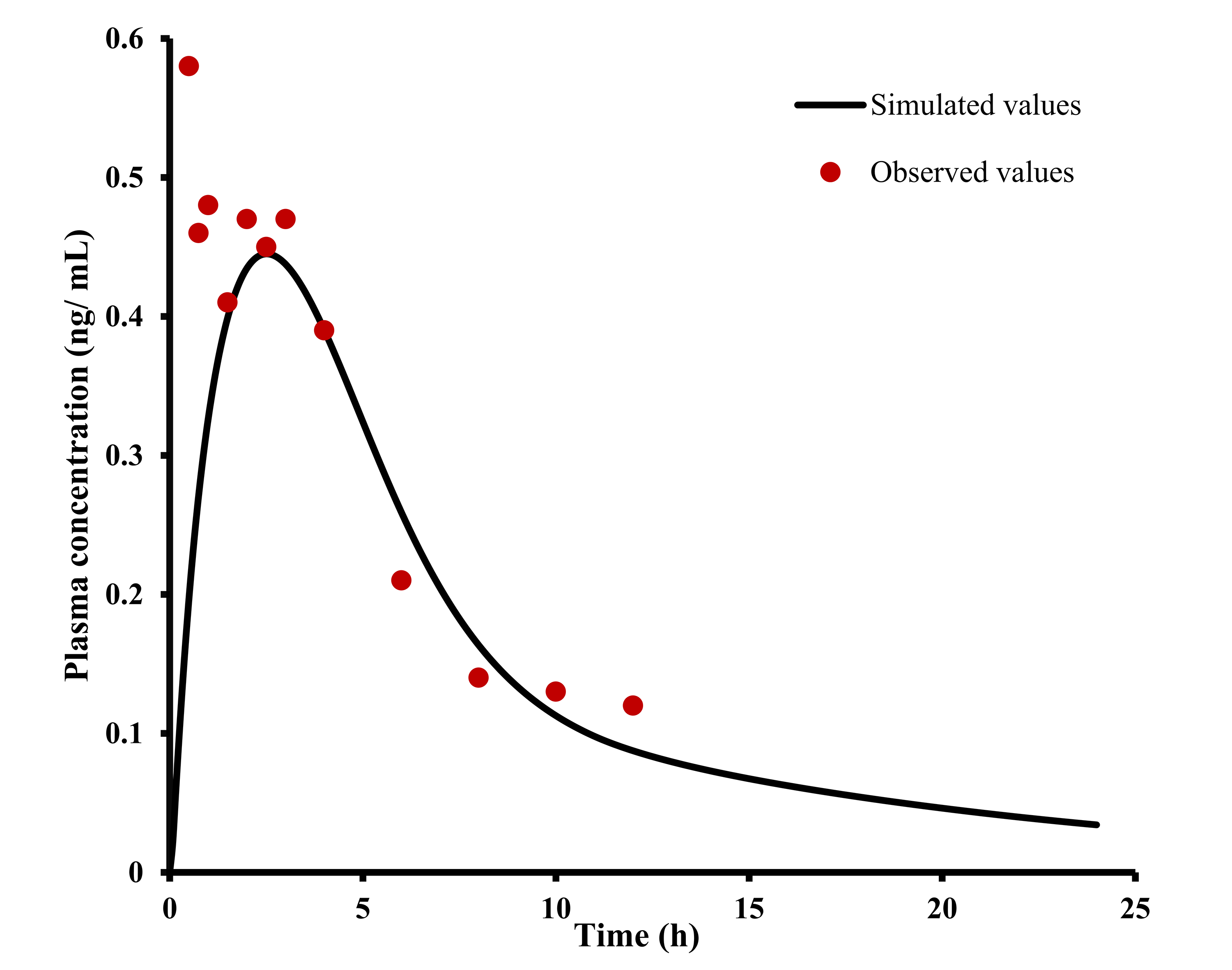

Supplement: Supplementary file 1 [file ijms-25-03047-s001.zip › Figure S2.tif]
